# Vitamin *D* Supplementation for Prevention of Dental Implant Failure: A Systematic Review

**DOI:** 10.1155/2022/2845902

**Published:** 2022-01-12

**Authors:** Alaa Makke

**Affiliations:** Oral and Maxillofacial Department, Umm AlQura University, Faculty of Dentistry, Makkah 24225, Saudi Arabia

## Abstract

**Background:**

Many factors play a significant role in osseointegration and healing after dental implant insertion and restoration. Some factors are related to dental biomaterials, such as the dental implant, prosthesis, and grafting materials. Other factors can be connected to operator skills and accumulated experience. Local and systemic patient-related factors are crucial in determining the success of the dental implant. Thorough examination and analysis of local factors using available examination tools are vital to prepare the implant candidate for such treatment. The patient's systemic condition directly affects the healing of the dental implant. One of the most overlooked systemic factors is the patients' vitamin *D* level, which influences bone formation around the implant and subsequent osseointegration. The current review examined the available literature regarding the association between vitamin *D* supplementation and dental implant osseointegration.

**Methods:**

Data of this review were derived from recent research available on PubMed, Google Scholar, and Scopus. Inclusion criteria were the relation between the vitamin *D* serum and dental implant osseointegration or failure. The Systematic Reviews and Meta-Analyses (PRISMA) checklist was followed to perform the review. The study's outcome was the need for vitamin *D* supplementation to prevent implant failure.

**Results:**

Five human studies (including case reports, case series, and retrospective studies) and six animal studies. All included studies discussed the relationship between vitamin *D*, early dental implant failure, and bone implant contact. Three retrospective studies found no significant relationship between vitamin *D* supplementation and EDIFs in humans. On the other hand, one retrospective study showed a significant relationship in humans. A case report and case series claimed that the implant was successfully placed after vitamin *D* supplementation. A total of four animal studies showed a significant relationship between vitamin *D* supplementation and osseointegration of the dental implant. Two animal studies showed no significant association.

**Conclusion:**

To ensure optimal treatment outcomes, it is recommended to supplement the patient with vitamin *D* if the serum level is not within the normal range. Further clinical studies and case reports are needed to confirm the association between serum vitamin *D* levels and osseointegration.

## 1. Introduction

Osseointegration is the key to dental implant success and is required to achieve direct structural and functional bone formation. For this to occur, many integrated factors should be thoroughly assessed, such as surgical techniques, type of prosthesis, biomaterials, and operator- and patient-related factors [1]. Implant failures are classified according to the timing of the loss to early dental implant failure (EDIF) or late dental implant failure (LDIF). Early implant failure occurs due to improper implant placement and restoration, low bone volume or density, systemic conditions, or smoking [2, 3].

The primary goal for any oral implantologist is to obtain satisfactory healing and long-term treatment success. Most researchers and manufacturing companies pursue innovations through new implant designs and surface treatment to increase stability and accelerate the osseointegration process. Often, systemic health is a significant factor that is not adequately considered. The human skeleton derives its vitality from key minerals, such as calcium, fluoride, magnesium, potassium, vitamin B6, vitamin *D*, and zinc [4]. The European Register of Nutrition and Health described 18 items that directly affect bone and teeth, including vitamin *D* [5], one of the most necessary micronutrients for dental implant osseointegration. Therefore, adequate exposure to sunlight and optimal dietary habits support the maintenance of satisfactory vitamin *D* levels [6].

Vitamin *D* insufficiency is defined as any serum level ranging between 21 and 29 ng/ml, deficiency is when levels are less than 20 ng/ml, and severe deficiency is less than 10 ng/ml (Table 1). Vitamin D3 is the basic form of the vitamin *D* family, and it is activated by hydroxylation in the liver. Vitamin *D* stimulates osteoclastic activity and the production of extracellular matrix proteins by osteoblasts. Moreover, it increases calcium absorption in the intestines [7–11]. Some studies proposed that a low vitamin *D* level might be associated with EDIF [2, 12]. Therefore, the purpose of the current review is to investigate the available literature regarding vitamin *D* supplementation and dental implant osseointegration.

## 2. Materials and Methods

The most recently published English research was reviewed, including animal studies, in vitro studies, case series, case reports, cohort studies, and clinical studies. The Preferred Reporting Items for Systematic Reviews and Meta-Analyses (PRISMA) checklist was followed to perform the review. Data were collected using the following keywords: vitamin *D*, dental implant, implant failure, osseointegration. A database such as Google Scholar, PubMed, Scopus, or ResearchGate was used to explore recent research. Inclusion criteria were studies investigating the vitamin *D* serum and dental implant osseointegration or failure. Exclusion criteria were incomplete data and irrelevant or duplicated studies. Data were reviewed according to the patient age and sex of the patient, the year of the study, and the number and location of the study.

## 3. Role of Vitamin *D* on Dental Implant Osseointegration

Sunlight vitamin *D* synthesis begins from cholesterol in the skin converted into pre-vitamin D3 and then isomerized to vitamin D3. Subsequently, 25-hydroxyvitamin D3 resulting after vitamin D3 is hydrolyzed in the liver [13]. Primarily, vitamin *D* stimulates the osteoclastic activity and production of extracellular matrix proteins by osteoblasts and increases the Ca absorption in the intestines [7–11]. Likewise, there is a microbicidal effect on monocytes affected by vitamin *D* levels, which increase the body's resistance to bacteria, fungi, and viruses [14]. There was a significant correlation between infection rate and vitamin *D* levels <20 ng/ml [15]. Therefore, the optimal vitamin *D* level might be one of the most critical systemic factors in preventing infection and providing an optimal environment for successful osseointegration.

## 4. Retrospective Studies and Case Reports

A retrospective study on 1,740 implants placed in 885 patients showed no significant relationship between low vitamin *D* levels and increased risk of EDIFs [16]. In patients with serum levels of vitamin *D* < 10 ng/ml, there were 11.1% EDIFs before loading the prosthetic part. In comparison, when the serum level of vitamin *D* was between 10 and 30 ng/ml, the EDIFs was 4.4%, and when the serum level of vitamin *D* was >30 ng/ml, there was a 2.9% EDIF rate. The retrospective study by Wagner et al. showed a significant and positive effect of vitamin *D* on marginal bone loss at the mesial and distal aspects of the dental implants [17].

Similarly, a retrospective study of 1,625 implants placed in 822 patients showed an increase in the incidence of EDIFs in patients with compromised vitamin *D* levels, but statistically, it was not significant. The study described 9% EDIF for those with a serum level of vitamin *D* < 10 ng/ml and 3.9% EDIFs for those with a serum level of vitamin *D* between 10 and 30 ng/ml. Surprisingly, a 2.2% EDIF rate was noted in patients with a serum level of vitamin *D* > 30 ng/ml [18].

A case series examined implants that were successfully osseointegrated in two patients with a history of EDIFs after vitamin *D* supplements [19]. Another case report claimed EDIFs occurred after immediate implant placement due to severe vitamin *D* deficiency [20] (Table 2).

## 5. Animal Studies

Kelly et al. found exposed implant surfaces in vitamin D-deficient rats and bone fracture between the implant and surrounding bone after 14 days of implant placement when subjected to a lower push-in test and lower bone to implant contact (BIC) [21]. On the contrary, vitamin *D* supplementation increased bone density by 1.2-fold and osseointegration by 1.5-fold and extrusion force resistance by 2.0-fold in osteoporotic rats. The trabecular bone amount was increased by 96%, and osseointegration was elevated up to 94.4%, while the trabecular number was elevated by 112.5% and 51.8% in connective tissue [22].

Wu et al. used vitamin *D* and insulin on diabetic rats to improve osseointegration. The study claimed that bone volume, osseointegration, trabecular bone number and thickness, connective tissue density, BIC, and push-out force were enhanced [23]. Another study on diabetic rats showed no significant effect of vitamin *D* on osseointegration. In a different study, vitamin *D* combined with insulin was administered to diabetic rats, and histological analysis was conducted during the third week after implant placement. A BIC level of 44 was measured in the diabetic rats and 57 for rats in the vitamin *D* + insulin group. Six weeks later, the BIC level was 70 for the control group and 65 for the vitamin *D* group 5 [24]. Dvorak et al. observed no significant rescission of BIC in the medullar and periosteal compartment among rats that received vitamin *D* in their diet compared to those which did not receive it [25].

A significant difference was observed when vitamin *D* supplements were administered to rats with chronic kidney disease (CKD) compared to those with CKD without supplementation. BIC levels were significantly higher among the supplementation group [26] (Table 3).

## 6. Discussion

The current review aimed to investigate the association between vitamin *D* levels and the osseointegration process of a dental implant. The United States Centers for Disease Control and Prevention's National Health and Nutrition Survey claimed an insufficient intake of vitamin *D* for more than 25% of the US population. This deficiency is shared outside the US, with more than 2 billion people worldwide also showing vitamin *D* deficiency. Thus, vitamin *D* deficiency directly affects the skeleton of the human body, including the jawbone [27], and therefore, it may play a critical role in dental implant treatment outcomes. Accordingly, a stable vitamin *D* level will assist in maintaining a healthy body, and ideal daily amounts are 2,000 IU (50 mcg) for most people and up to 4,000 IU [27] during pregnancy.

The current review included five human studies (including case reports, case series, and retrospective studies) and six animal studies. All included studies discussed the relationship between vitamin *D*, EDIFs, and BIC.

A significant relationship was found between vitamin *D* and osseointegration in four animal studies when BIC was examined. A lower push-in test was performed 14 days after implant insertion in rats. Kelly et al. found that, after scanning electron microscopy (SEM) analysis, all rats with vitamin *D* deficiency showed exposed implant surfaces, which occurred because of bone fracture between the implant and the surrounding bone [21]. Likewise, an investigation conducted to determine the effect of 1,25(OH)2D3 on implant osseointegration in osteoporotic rats indicated that vitamin *D* supplementation resulted in significant bone formation around the implant [22].

Other animal studies showed that bone volume increased when vitamin *D* was used with insulin in diabetic rats after implant placement [24]. The mean bone volume and osseointegration outcomes of the studies were very similar to those of healthy rats. Similarly, Liu et al. proved that CKD rats that received vitamin *D* supplementation showed greater BIC and bone volume than CKD rats that did not receive vitamin *D* supplementation [23]. The studies, as mentioned earlier, showed the importance of vitamin *D* supplementation, especially for compromised rats. The findings of these studies demonstrated the significance of supplemental vitamin *D* in the short- and long-term outcomes after dental implant treatment.

Two animal studies showed no significant correlation between vitamin *D* supplementation and dental implant osseointegration [24, 25]. Akhavan et al. showed that vitamin *D* supplementation did not significantly affect osseointegration, although the study did not mention if the experimental rats received diabetes medication [24]. Dvorak et al. showed that there no significant difference when rats were fed a standard vitamin *D* diet [25]. However, the study did not mention the vitamin *D* blood levels of the rats after diet administration.

There has been a lack of human studies examining the association between vitamin *D* serum levels and osseointegration. In 2018, Mango et al. investigated vitamin *D* serum levels and EDIFs in a retrospective clinical study that included 885 patients, with specified inclusion and exclusion criteria [16]. Their findings showed 35 EDIFs (3.9%) reported, with no significant relationship between the EDIFs and patient gender, smoking, history of periodontal disease, or vitamin deficiency. Three EDIFs were noted in 27 patients with vitamin *D* serum levels <10 ng/ml, 20 EDIFs in 448 patients with groups of 10–30 ng/ml, and 12 EDIFs in 410 patients with serum levels >30 ng/ml. Limitations of the study included the sample size of the patients with low serum levels of vitamin *D* and the retrospective design.

One case report found severe vitamin *D* deficiency correlated with immediate implant placement [20]. A case series confirmed this hypothesis when the implant was successfully osseointegrated after *D* supplementation, although there was a previous implant failure [19]. Interestingly, the results of the retrospective studies, case reports, and case series concluded that the serum level of vitamin *D* seems to influence dental implant osseointegration. The limitation of the study includes a limited number of available data and sample size and few clinical studies.

## 7. Conclusions

Additional prospective clinical studies and case reports are required to determine the association between serum vitamin *D* levels and osseointegration. Some studies showed a clear association in terms of osseointegration and EDIFs. Additional studies with specific inclusion and exclusion criteria are necessary to isolate vitamin *D* levels as the only factor tested. If a patient's vitamin *D* level is severely compromised according to the level mentioned in this review, the recommendation is to supplement with vitamin *D* to ensure optimal treatment outcomes.

## Figures and Tables

**Table 1:** Vitamin *D* levels.

*Serum 25(OH) vitamin D levels*	
Optimal	30 ng/mL
Insufficiency	10–30 ng/mL
Deficiency	10 ng/mL

**Table 2:** Data from case reports, case series, and retrospective studies.

Author	Title	Study type	Results
Mango et al., 2018	Low serum level vitamin *D* and early dental implant failure	Retrospective study	No significant relationship between low vitamin *D* levels and an increase in the risk of EDIF

Wagner et al., 2017	Does osteoporosis influence the marginal peri-implant bone level in female patients? A cross-sectional study in a matched collective	Retrospective parallel-group	A significant and positive effect of vitamin *D* on marginal bone loss at the *M* and *D* aspect of the dental implants

Mangano et al., 2016	Is low serum vitamin *D* associated with early dental implant failure? A retrospective evaluation of 1625 implants placed in 822 patients	Retrospective study	Increase in EDIF with compromised levels of vitamin *D*, but statistically not significant

Fretwurst et al., 2017	Vitamin *D* deficiency in early implant failure: Two case reports	Case series	The implant was successful after administration of vitamin D

Bryce and macbeth, 2014	Vitamin *D* deficiency as a suspected causative factor in the failure of an immediately placed dental implant: A case report	Case report	The EDIFs occurred after immediate implant placement due to severe vitamin *D* deficiency

**Table 3:** Data from animal studies.

Author	Title	Study type	Results
Kelly et al., 2008	Vitamin *D* and bone physiology: Demonstration of vitamin *D* deficiency in an implant osseointegration rat model	Animal study	Exposed implant surface in vitamin D-deficient rats

Zhou et al., 2012	1,25-Dihydroxy vitamin D3 improves titanium implant osseointegration in osteoporotic rats	Animal study	Increased bone density by 1.2-fold, osseointegration by 1.5-fold, and extrusion force resistance by 2.0-fold in osteoporotic rats. The trabecular bone amount was elevated by 96%, and osseointegration was elevated up to 94.4%. Trabecular number was elevated by 112.5% and 51.8% connective tissue

Wu et al., 2012	Vitamin D3 and insulin combined treatment promotes titanium implant osseointegration in diabetes mellitus rats	Animal study	Bone volume, osseointegration, trabecular bone number and thickness, connective tissue density, BIC, and push-out force were improved

Akhavan et al., 2012	The effect of vitamin *D* supplementation on bone formation around titanium implants in diabetic rats	Animal study	No significant effect of vitamin *D* on osseointegration in diabetic rats

Dvorak et al., 2012	Impact of dietary vitamin *D* on osseointegration in the ovariectomized rat	Animal study	No significant rescission of the BIC in the medullar and periosteal compartment among rats that received vitamin *D* when compared to those that did not

Liu et al., 2014	Vitamin *D* supplementation enhances the fixation of titanium implants in CKD mice	Animal study	A significant difference was observed when vitamin *D* supplements were given to rats with CKD

## Data Availability

The data supporting this review are from previously reported studies and datasets, which have been cited.

## Abbreviations

BIC: Bone to implant contact
EDIFs: Early dental implant failures.

## References

[1] Chrcanovic B. R., Kisch J., Albrektsson T., Wennerberg A. (2018). A retrospective study on clinical and radiological outcomes of oral implants in patients followed up for a minimum of 20 years. *Clinical Implant Dentistry and Related Research*.

[2] Mangano F. G., Mastrangelo P., Luongo F., Blay A., Tunchel S., Mangano C. (2017). Aesthetic outcome of immediately restored single implants placed in extraction sockets and healed sites of the anterior maxilla: a retrospective study on 103 patients with 3 years of follow-up. *Clinical Oral Implants Research*.

[3] Buhara O., Pehlivan S. (2018). Estimating the importance of significant risk factors for early dental implant failure: a Monte Carlo simulation. *The International Journal of Oral & Maxillofacial Implants*.

[4] Javed F., Malmstrom H., Kellesarian S. V., Al-Kheraif A. A., Vohra F., Romanos G. E. (2016). Efficacy of vitamin D3 supplementation on osseointegration of implants. *Implant Dentistry*.

[5] Iolascon G., Gimigliano R., Bianco M. (2017). Are dietary supplements and nutraceuticals effective for musculoskeletal health and cognitive function? A scoping review. *The Journal of Nutrition, Health & Aging*.

[6] Nastri L., Moretti A., Migliaccio S. (2020). Do dietary supplements and nutraceuticals have effects on dental implant osseointegration? A scoping review. *Nutrients*.

[7] Brinker M. R., O’Connor D. P., Monla Y. T., Earthman T. P. (2007). Metabolic and endocrine abnormalities in patients with nonunions. *Journal of Orthopaedic Trauma*.

[8] Alkalay D., Shany S., Dekel S. (1989). Serum and bone vitamin D metabolites in elective patients and patients after fracture. *Journal of Bone and Joint Surgery British Volume*.

[9] DeLuca H. F. (1988). The vitamin D story: a collaborative effort of basic science and clinical medicine 1. *The FASEB Journal*.

[10] Bouillon R., Okamura W. H., Norman A. W. (1995). Structure-function relationships in the vitamin D endocrine system. *Endocrine Reviews*.

[11] Christakos S., Dhawan P., Liu Y., Peng X., Porta A. (2003). New insights into the mechanisms of vitamin D action. *Journal of Cellular Biochemistry*.

[12] Dusso A. S., Brown A. J., Slatopolsky E. (2005). Vitamin D. *American Journal of Physiology - Renal Physiology*.

[13] Apostu D., Lucaciu O., Lucaciu G. D. O. (2017). Systemic drugs that influence titanium implant osseointegration. *Drug Metabolism Reviews*.

[14] Lehmann B. (2005). The vitamin D3 pathway in human skin and its role for regulation of biological processes. *Photochemistry and Photobiology*.

[15] Liu P. T., Stenger S., Li H. (2006). Toll-like receptor triggering of a vitamin D-mediated human antimicrobial response. *Science*.

[16] Mango F., Osakoui S., Paz A., Mango N., Manago C. (2018). Low serum level Vitamin D and early dental implant faliure. *Joddd*.

[17] Wagner F., Schuder K., Hof M., Heuberer S., Seemann R., Dvorak G. (2017). Does osteoporosis influence the marginal peri-implant bone level in female patients? A cross-sectional study in a matched collective. *Clinical Implant Dentistry and Related Research*.

[18] Mangano F., Mortellaro C., Mangano N., Mangano C. (2016). Is low serum vitamin D associated with early dental implant failure? A retrospective evaluation on 1625 implants placed in 822 patients. *Mediators of Inflammation*.

[19] Fretwurst T., Grunert S., Woelber J. P., Nelson K., Semper-Hogg W. (2016). Vitamin D deficiency in early implant failure: two case reports. *International Journal of Implant Dentistry*.

[20] Bryce G., MacBeth N. (2014). Vitamin D deficiency as a suspected causative factor in the failure of an immediately placed dental implant: a case report. *Journal of the Royal Naval Medical Service*.

[21] Kelly J., Lin A., Wang C. J., Park S., Nishimura I. (2009). Vitamin D and bone physiology: demonstration of vitamin D deficiency in an implant osseointegration rat model. *Journal of Prosthodontics*.

[22] Zhou C., Li Y., Wang X., Shui X., Hu J. (2012). 1,25Dihydroxy vitamin D3 improves titanium implant osseointegration in osteoporotic rats. *Oral Surgery, Oral Medicine, Oral Pathology and Oral Radiology*.

[23] Wu Y.-y., Yu T., Yang X.-y. (2013). Vitamin D3 and insulin combined treatment promotes titanium implant osseointegration in diabetes mellitus rats. *Bone*.

[24] Akhavan A., Noroozi Z., Shafiei A., Haghighat A., Jahanshahi G., Mousavi S. (2012). The effect of vitamin D supplementation on bone formation around titanium implants in diabetic rats. *Dental Research Journal*.

[25] Dvorak G., Fügl A., Watzek G., Tangl S., Pokorny P., Gruber R. (2012). Impact of dietary vitamin D on osseointegration in the ovariectomized rat. *Clinical Oral Implants Research*.

[26] Liu W., Zhang S., Zhao D. (2014). Vitamin D supplementation enhances the fixation of titanium implants in chronic kidney disease mice. *PLoS One*.

[27] Isaia G., Giorgino R., Rini G. B., Bevilacqua M., Maugeri D., Adami S. (2003). Prevalence of hypovitaminosis D in elderly women in Italy: clinical consequences and risk factors. *Osteoporosis International*.

